# Enhancing hydrophobicity, strength and UV shielding capacity of starch film via novel co-cross-linking in neutral conditions

**DOI:** 10.1098/rsos.181206

**Published:** 2018-11-14

**Authors:** Ni Shuzhen, Jiao Liang, Zhang Hui, Zhang Yongchao, Fang Guigan, Xiao Huining, Dai Hongqi

**Affiliations:** 1 Jiangsu Co-Innovation Center of Efficient Processing and Utilization of Forest Resources, Nanjing Forestry University, Nanjing 210037, People's Republic of China; 2 College of Materials Engineering, Fujian Agriculture and Forestry University, Fuzhou 350002, People's Republic of China; 3 Department of Chemical Engineering, University of New Brunswick, Fredericton, New Brunswick, Canada E3B 5A3; 4 Johan Gadolin Process Chemistry Centre, c/o Laboratory of Wood and Paper Chemistry, Åbo Kademi University, Turku 20500, Finland

**Keywords:** starch film, co-cross-linking, neutral condition, strength, hydrophobicity, transmittance

## Abstract

Starch films are developed as the biodegradable packaging materials to replace the petroleum-based materials in recent years. Thus, it is extremely beneficial to improve the hydrophobicity and mechanical strength of starch films, through a novel approach of co-cross-linking in neutral conditions, with glyoxal and AZC. In this work, systematic studies have been conducted to assess the performance of the co-cross-linked starch along with the control starch and starch cross-linked by glyoxal or AZC alone. Results showed that the co-cross-linked starch films exhibited significantly improved hydrophobicity and strength and the wet stress reached 1.53 MPa, compared to the control, glyoxal or AZC cross-linked starch films. More interestingly, the co-cross-linked film also demonstrated excellent UV shielding capacity and transmittance at visible wavelength range. The reaction mechanism was revealed based on the findings from UV, FT-IR and NMR spectra. This work established an innovative approach to improving the performance of starch film in neutral conditions for packaging applications.

## 1. Introduction

Starch, a category of non-toxic, biodegradable and low-cost polysaccharide, has been widely applied to many fields and attracts a great deal of attention as a potential alternative to the conventional petroleum-based materials [1–4]. In particular, its utilization in the packaging industry promotes sustainability and addresses the negative impact that non-biodegradable plastics have on the environment [5,6]. To meet the requirements for the packaging materials, it is highly necessary to improve the hydrophobicity and strength of various forms of starch-based materials including films, foam and starch-coated papers [7–9]. Therefore, much attention is paid to improving the hydrophobicity and mechanical properties of starch.

Cross-linking can link two polymer chains by bi-functional reagents, by reducing the water-binding capacity and enhancing the stress-transferring ability. As a popular approach, the formed covalent linkages among the molecular chains can efficiently limit the movement of polymer chains and improve the performance [10–12]. In the papermaking industry, it is commonly used to improve the water resistance and strength of starch-coated papers in various ways, including the addition of moisture proof agents or cross-linking agents in the starch solution, such as glutaraldehyde (GA), formaldehyde (FA), butanetetracarboxylic acid (BTCA), citric acid (CA), etc. [13–16].

However, a greener and economically more efficient approach is expected with enhanced environmental awareness in order to avoid the release and utilization of the hazardous chemicals. Glyoxal is a bi-aldehyde cross-linking agent that has been approved by the FDA for use in the food packaging industry and can be linked to starch molecular chains on two ends. Besides, the relatively low temperature of the cross-linking process induced by glyoxal is an overwhelming advantage over the carboxylic cross-linking agents which often need temperatures as high as 170°C [17]. Based on this, glyoxal has been widely applied to cross-link the cellulose or starch-based materials, through the acetal linkages between the aldehyde and hydroxyl groups [18–23]. Furthermore, as glyoxal is used to cross-link polymers like chitosan and protein, which contain amino groups, a Schiff-base reaction between the aldehyde and hydroxyl groups might also contribute to improving the polymer performance [24,25].

Considering the versatility of glyoxal, a novel co-cross-linking system based on glyoxal was developed in this work. Ammonium zirconium carbonate (AZC) is a water-soluble inorganic salt that has been widely used to improve the water resistance of coated papers or enhance the performance of the xylan, galactoglucomannan and starch films via the hydrogen linkages between Zr and hydroxyl groups [26–28]. Besides, AZC has been also approved by the FDA to be used in the food packaging fields [29]. However, it was reported that the self-polymerization of AZC tends to weaken the performance of the starch along with the release of ammonia during the cross-linking process.

**Figure 1.**
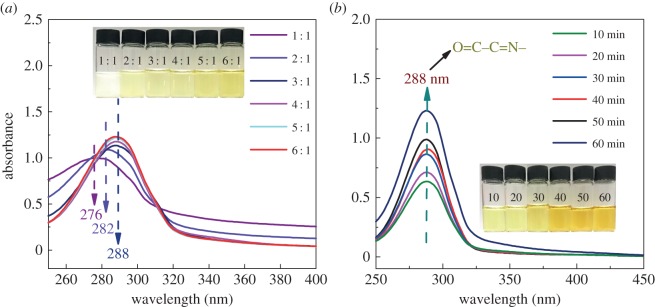
(*a*) The effect of the molar ratio of glyoxal/AZC and the heating time (*b*) with the corresponding molar ratio fixed at 6 : 1 on the UV–vis absorbance of the mixture solution of glyoxal and AZC heated at 60°C for 30 min. The AZC concentration in the mixture solution is 0.25% (wt) of starch.

In our work, we found the addition of glyoxal can limit the self-polymerization of AZC in aqueous solution (in electronic supplementary material, figure S1 and figure 1). Therefore, the co-cross-linking system of glyoxal and AZC in neutral conditions is proposed. The Schiff-base reaction between glyoxal and NH_3_ might also improve the UV shielding ability of co-cross-linked starch film, which can avoid the oxidation of lipid and prolong the shelf life of packaged food. Hence, it is significant to systematically study the strength, hydrophobic properties and transmittance of starch films produced via this co-cross-linking system.

In this present work, the reaction mechanism of the starch co-cross-linking created by glyoxal and AZC (Gly–AZC) was first revealed with UV–vis, FT-IR spectroscopy and ^13^C NMR. Subsequently, XRD and TG characterizations were used to confirm that the glyoxal can efficiently limit the self-polymerization of AZC. Lastly, the hydrophobicity, mechanical and optical properties of Gly–AZC films were evaluated, i.e. the water stability, swelling ratio, contact angle, transmittance and tensile strength. Meanwhile, the performance of co-cross-linked starch film was compared with that of the control starch film, glyoxal or AZC cross-linked starch film alone. The findings from the current work will be helpful in providing a novel modification method for broadening the application fields of the starch-based packaging films.

## 2. Material and methods

### 2.1. Materials

Soluble starch (MW 342) and AZC with ZrO content of 18% were purchased from Guoyao Chemical Co., Ltd., China. Glyoxal water solution (40%) was kindly provided by Nanjing Chemical Industry Group (China). All chemicals were used without any further purification.

### 2.2. Methods

#### 2.2.1. Fabrication of cross-linked starch films

The 10% control starch solutions were prepared by dissolving 10 g soluble starch into 90 g water through heating at 90°C for 2 h. The Gly–AZC starch solution was obtained through first heating at 60°C for 1 h and then heating at 90°C for another 1 h. The dosages of glyoxal and AZC were 10% (wt) and 5% (wt) to the dry starch, respectively. For comparison, the starch solution added by glyoxal or AZC alone was also prepared under the same condition, at a dosage of 15%. The as-prepared starch solution was poured into the hydrophobic Petri dishes and oven-dried at 40°C for 2 h. Before characterization, the films were kept at 23°C and a relative humidity (RH) of 50% for 2 days and then peeled off. The grammage of films was determined based on the amount of dry starch (g) over the unit area coated. The starch granules for the characterizations of FT-IR, NMR, TGA and XRD were obtained through grinding the starch samples using a solid grinder (FW100, Tianjin Taisite Corp, China) for 5 min after the as-prepared starch solutions were freeze-dried at −80°C and 15 Pa for 48 h.

#### 2.2.2. Characterization

The water contact angle of starch film was measured by the sessile drop method using a contact angle analyser (Attention Theta, Biolin Scientific Corp, Sweden) at 25°C and 50% RH with 4 µl of deionized water [30]. The reported values were measured at 15 s after the deposition of droplets and every sample was performed 10 times in different positions.

For the testing of mechanical properties, the starch films were cut into 5 mm × 30 mm rectangular strips. Afterwards, their tensile stress and Young's modulus were measured at room temperature using a UTM6502 universal testing machine (Suns, Shenzhen, China) equipped with a 500 N load cell and pneumatic grips. The cross-head speed during tensile testing was 5 mm min^−1^. The specific tensile stress and specific Young's modulus were calculated accordingly [31].

The water stability tests were conducted as follows. The starch films were immersed in deionized water and stirred for 5 min (600 rpm) at 25°C and 60°C, respectively. Then the photographs were taken to determine the water stability of starch films in water.

The swelling ratio of control and cross-linked starch film in water was also determined. Firstly, the cross-linked starch films were conditioned at 105°C for 4 h to remove the moisture and weighted as *M*_1_. Then the films were soaked in deionized water for 7 min and the swollen films were wiped gently using Kimwipes every minute and weighed as *M*_2_. The swelling ratio of starch films in water was calculated as follows:

2.1 $$\text{Swelling ratio}\;(\text{\%})=\frac{M_{2}-M_{1}}{M_{1}}\times 100\text{\%}.$$

A UV–Vis spectrometer (TU-1900, Beijing Purkinje Corp., China) was used to characterize the light transmittance of the as-prepared starch films and record the adsorption of the reacted mixture solution of glyoxal and AZC from 200 to 800 nm, in accordance with the procedures in related literature [32–34].

The 650 FT-IR spectrometer (Tianjin GangDong Sci & Tech. D) was used on the control starch, glyoxal cross-linked starch, AZC cross-linked starch and Gly–AZC starch granules after grinding with KBr by co-adding over 16 scans, capturing spectra between 500 and 4000 cm^−1^, at a resolution of 0.5 cm^−1^. ^13^C NMR of the starch granules dissolved in deuterium peroxide (D_2_O) were recorded on a Varian Unity 400 spectrometer operated at 400 MHz.

The thermal properties of the starch were examined using an SDT Q600 TGA System thermogravimetric analyser (TA, America). The sample weight was 1–10 mg and heated from 30°C to 800°C under the nitrogen atmosphere at a heating rate of 10°C/min and a nitrogen flow rate of 100 ml min^−1^.

The Ultima X-ray diffractometer (Rigaku, Japan) was used to determine the crystallinity of starch samples over a 2*θ* range from 5° to 50° with a scan step time of 30 s.

The surface of starch granules and the cross-sections of the starch films were observed using a Quanta 200 Environmental scanning electron microscope (FEI, America) after gold sputtering according to the reference [35]. The starch films were immersed in liquid nitrogen before fracturing and the starch granules were obtained using a solid grinder (FW100, Tianjin Taisite Corp, China) after freeze-drying. The surface topographical images of starch films were also examined using a Dimension Edge Atomic Force Microscope (Bruck, Germany) in tapping mode at 300 kHz with the starch film stuck on a silicon cantilever.

## 3. Results

### 3.1. UV–vis spectra of the mixture solution of glyoxal and AZC

The adsorption of the as-prepared mixture solution of glyoxal and AZC was recorded with the UV–vis spectra, and the results are presented in figure 1. As depicted, the maximum adsorption wavelength of mixture solution shifts from 276 to 288 nm as the molar ratio of glyoxal to AZC varies from 1 : 1 to 6 : 1. According to our previous work (electronic supplementary material, figure S1), the glyoxal solution and AZC solution heated at 60°C for 30 min appeared to be transparent and milky white, respectively; and such adsorption intensity was not observed around 288 nm in the UV–vis spectra. The ‘white’ colour in the AZC solution indicates the self-polymerization of AZC upon heating. Here we found that the solution mixture of glyoxal and AZC changes from white to light yellow with the increasing molar ratio in figure 1*a*. When the molar ratio rises to 2 : 1, the solution becomes clear and transparent without any precipitation. This can be attributed to the fact that the increased glyoxal dosage efficiently limits the self-polymerization of AZC through the Schiff-base reaction with AZC.

As the molar ratio increases, an obviously enhanced adsorption intensity appears at 282 nm due to the newly produced Schiff-base structure (O = C–C = N), indicating the successful reaction between the aldehyde and amino groups. As the glyoxal dosage further increases, the maximum adsorption wavelength shifts to 288 nm and the solution becomes dark yellow. It is confirmed that the Schiff-base reaction between glyoxal and AZC successfully occurs, and the nitrogen element in the AZC can be retained. Besides, the exhibited prominent UV adsorbing ability of glyoxal–AZC solution might be beneficial for improving the UV shielding capacity of the co-cross-linked starch films.

Figure 1*b* illustrates the effect of reaction time on the UV–vis spectra of the mixture solution of glyoxal and AZC. As the reaction proceeds, the solution colour changes from light yellow to dark yellow; and enhanced absorbance intensity is observed, indicating an increased amount of Schiff-base structure. The turbidity phenomenon is not observed due to the polymerization of AZC, which indicates that the free ‘Zr’ in AZC have the chance to form the hydrogen linkage with the hydroxyl groups of starch. Thus, this result is extremely beneficial for increasing the cross-linking density of starch and improving its stress-transferring ability.

### 3.2. Cross-linking reactions via functional groups

**Figure 2.**
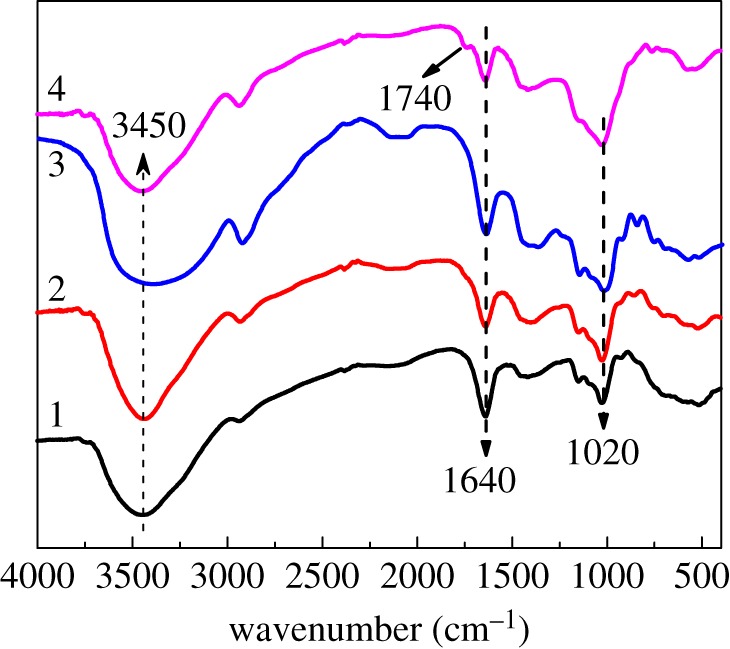
FT-IR spectra of control starch (Control, 1), glyoxal cross-linked starch (Glyoxal, 2), AZC cross-linked starch (AZC, 3), glyoxal and AZC co-cross-linked starch (Gly–AZC, 4).

**Figure 3.**
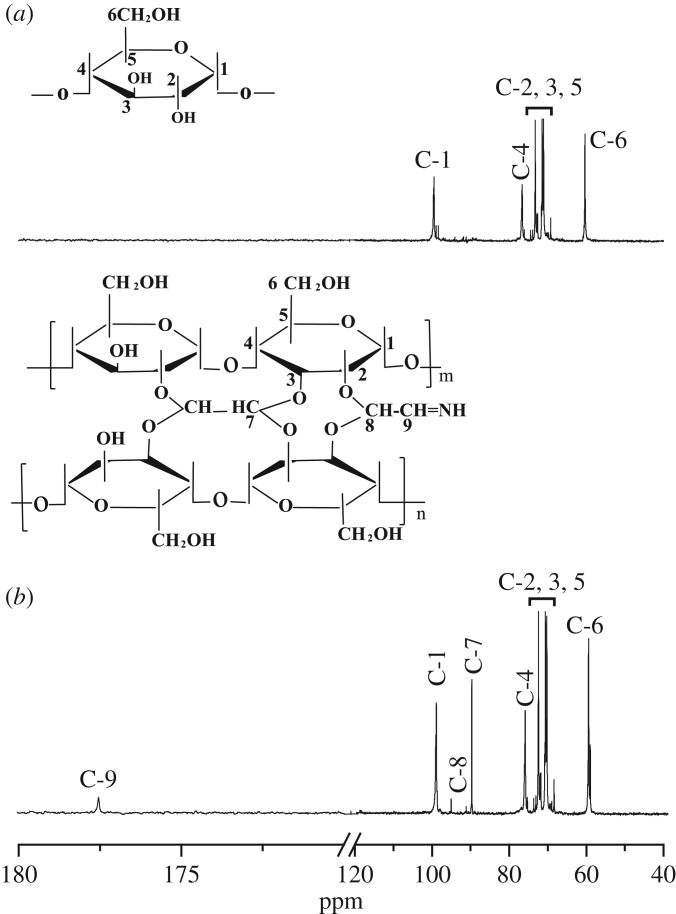
^13^C NMR spectra of control starch (*a*) and co-cross-linked starch by glyoxal and AZC (*b*).

To further confirm the co-cross-linking of starch induced by glyoxal and AZC, FT-IR spectra and ^13^C NMR of the starch samples were collected, and the results are shown in figures 2 and 3. As can be seen from FT-IR spectra, the adsorption peak of -OH around 3450 cm^−1^ in the AZC sample becomes broader compared with the other samples, which can be attributed to the re-association of hydroxyls caused by the hydrogen linkage between Zr and hydroxyl groups [36]. The adsorption peak at 1640 cm^−1^ can be ascribed to the stretching vibration of hydrogen bonds [37]. As reported, the cross-linking reaction between aldehyde and hydroxyl groups can decrease the amount of hydrogen bonds and give rise to the amount of C–O linkages. Therefore, the intensity at 1640 cm^−1^ is decreased, whereas the intensity at 1020 cm^−1^ is enhanced in Glyoxal and Gly–AZC samples. The adsorption peak at 1740 cm^−1^ in the Gly–AZC sample is attributed to the stretching vibration of O = C–C = N, indicating the successful Schiff-base reaction between glyoxal and NH_3_.

The chemical shifts and assignments of ^13^C NMR are given in figure 3. As shown in figure 3*a*, the signal for the C6-position of the starch backbone is observed at 60.2 ppm. The signals of C2-5 of the anhydroglucose appear between 69.3 ppm and 77.0 ppm. The signals at 99.6 ppm and 76.6 ppm are assigned to the C-1 and C-4, respectively. In figure 3*b*, the new signal of C-9 appeared at 177.5 ppm confirms that NH_3_ has been successfully covalently bonded to the glyoxal through the Schiff-base reaction. The signals of C-8 and C-7 at 95.4 ppm and 90.6 ppm are observed due to the acetalization between glyoxal and the hydroxyl groups of starch.

### 3.3. Crystallographic structures of control and cross-linked starch granules

**Figure 4.**
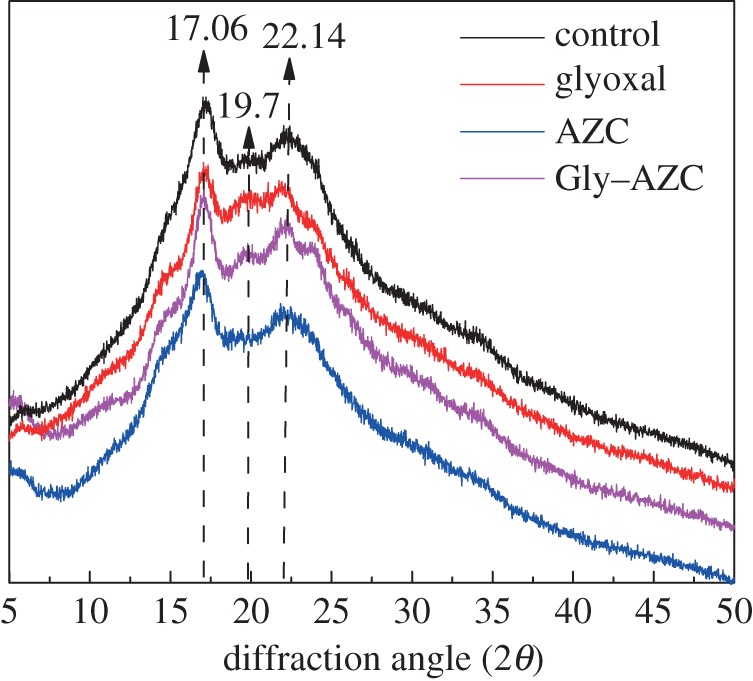
XRD spectra of control and cross-linked starch granules.

The X-ray diffraction patterns for both control and cross-linked starch granules are shown in figure 4. The starches exhibit two strong characteristic peaks at around 2*θ* value of 17.06° and 22.14° and a weak peak at near 2*θ* value of 19.7°. Compared with control starch, the AZC sample shows an obviously decreased intensity of reflection at 2*θ* = 19.7°, indicating the loss of ordered structure in starch. It is indicated that the AZC cross-linking with the starch molecules brings a significant reduction to the crystallinity of starch. This might be attributed to the decreased amount of the inter- and intra-molecular hydrogen bonds in the starch. By contrast, decreased crystallinity is not observed in the Gly–AZC sample which is further confirmation that the addition of glyoxal can efficiently limit the self-polymerization of AZC.

### 3.4. Thermal properties of starch granules

**Figure 5.**
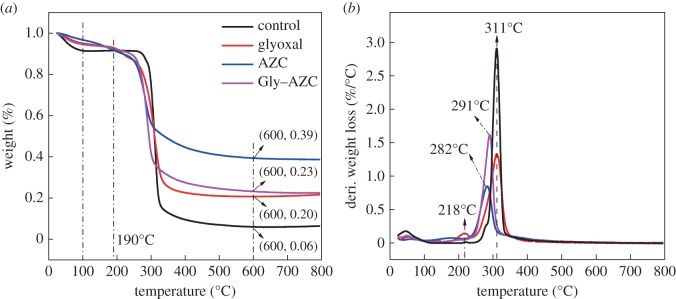
TGA (*a*) and DTG (*b*) curves for control and cross-linked starch granules.

Thermal properties of the control and cross-linked starches are characterized by thermogravimetric analysis (TGA). As shown in figure 5*a*, the control and cross-linked starch samples present two distinct stages. The first weight loss occurring between 100°C and 190°C is attributed to the loss of free and combined water in the starch granules. The cross-linking gives an obvious increase in the hydrophobicity of starch granules, and thus a lower mass loss of water is seen in the cross-linked starch samples.

In the second process, the initial decomposition temperature of the starch samples after cross-linking begins at 190°C; by contrast, the control starch is still stable even at around 300°C. This phenomenon might be related to the loose structure of starch due to cross-linking, which is accorded with the decreased crystallinity (figure 4). However, the decomposed mass of the cross-linked starch is much lower than that of control starch, which is also reflected in the DTG curves (figure 5*b*). Compared with the control starch, the intensity of cross-linked starch (Glyoxal, AZC and Gly–AZC) at the maximum degradation temperature is much lower. Besides, the maximum weight loss (MWS) peak in the AZC and Gly–AZC samples appeared at 282°C and 291°C, respectively, in comparison to that (311°C) of control starch. It seems that the aggregation of AZC created by its self-polymerization might destroy the ordered structure of the starch chain, thus resulting in the decreased maximum decomposition temperature (MDT); while the addition of glyoxal can limit the self-polymerization of AZC to some extent, thus giving a higher MDT than the AZC cross-linked starch. The thermal decomposition peak appearing in the Glyoxal sample at 218°C might be ascribed to the degradation of the volatile structure in the glyoxal cross-linked starch.

The cross-linking gives rise to the starch mass remaining after 600°C, as compared to the control starch (figure 5*a*). As seen, the residual contents in the AZC, Glyoxal and Gly–AZC samples at 600°C reach as high as 39, 23 and 20%, respectively, which is much higher than that of the control sample (6%). The results are accorded with the decreased decomposition weight loss at the corresponding MDT (figure 5*b*), indicating the improved thermal stability after cross-linking.

### 3.5. Water stability and water resistance of cross-linked starch films

**Figure 6.**
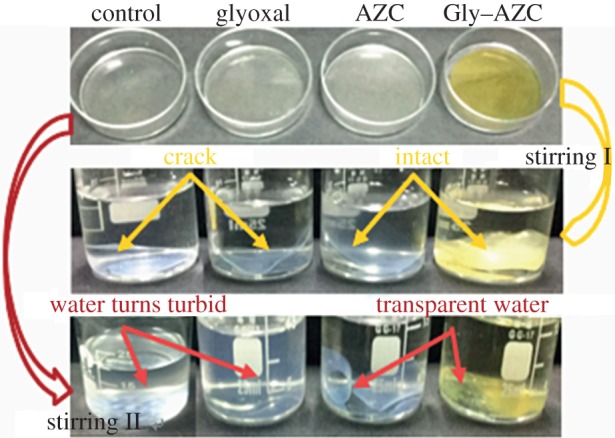
Stability of starch film samples in ambient water (stirring I, 25°C) and hot water (stirring II, 60°C) under magnetic stirring (600 rpm) for 10 min. The grammage of starch films is 200 g m^−2^.

Figure 6 shows the stability of the starch film in water at 25°C and 60°C under constant stirring. It is observed that an obvious crack appeared on the surface of the Control and Glyoxal sample after 10 min at 25°C (see Stirring I), while the AZC and Gly–AZC sample remains stable and keeps its intact original shape under the same conditions. It is indicated that the AZC cross-linking can effectively improve the water stability of the starch film. Because of the formation of the compact network structure after the cross-linking, the fractured regions previously accessible to water are reduced in the starch film completely or partly cross-linked by AZC. After stirring for 10 min at 60°C (see Stirring II), the Control sample disintegrates partly and the aqueous solution becomes turbid; while the Glyoxal sample still dissolves. This can be ascribed to the unreacted glyoxal in neutral conditions or the produced loose structure, thus water molecules move towards the inner parts of starch film readily and proceed with the disintegration of films, especially at a high temperature. The AZC and Gly–AZC films are curled, but still keep their original shape without any disintegration. It can be concluded that the water stability of the starch films cross-linked by glyoxal and AZC is significantly improved.

**Figure 7.**
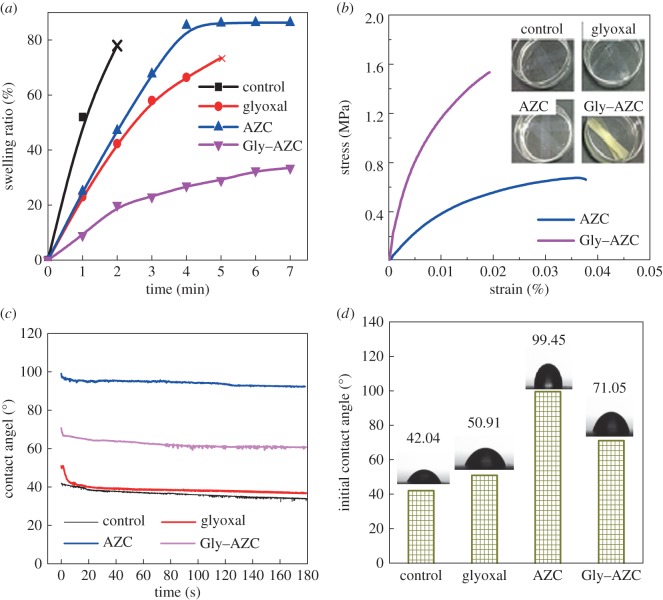
The swelling ratio of the starch films (*a*); the wet tensile stress of AZC and Gly–AZC starch films impregnated in water at 25°C for 2 h (*b*). The dynamic (*c*) and initial (*d*) water contact angle of starch films. The grammage of starch films is 140 g m^−2^.

Starch is an inherently hydrophilic polymer that can easily absorb moisture due to the presence of hydroxyl groups; but this feature weakens its mechanical properties. The swelling ratio of cross-linked starch films with different cross-linkers in water is shown in figure 7*a*. Swelling does occur after the diffusion of water within the starch polymer network. The cross-linking can lead to the formation of a compact and rigid network, which limits the absorption and diffusion of water molecules [38]. From figure 7*a*, it can be seen that the swelling ratios of the Glyoxal, AZC and Gly–AZC samples are decreased obviously compared with the Control sample, particularly in the first 2 min. Specifically, Glyoxal sample keeps intact shape until 5 min, while the AZC and Gly–AZC samples do not disintegrate even after 7 min. Meanwhile, the swelling ratio of Gly–AZC decreases obviously, compared with the other samples, indicating the formation of a highly cross-linked structure in the network induced by the novel co-cross-linking system consisting of glyoxal and AZC.

The above phenomenon can be clearly illustrated from the optical images of these four samples impregnated in water for 2 h at 25°C. As seen in figure 7*b*, the Control sample almost dissolves in water and presents a transparent state. The Glyoxal sample swells and dissolves partly, without sufficient wet-strength to keep its shape intact. By contrast, the AZC and Gly–AZC film is insoluble in water, suggesting the efficient strengthening of starch polymer networks caused by the cross-linking. The AZC presents a compact and intact shape without any sign of swelling and disintegration. Its wet tensile strength reaches to 0.66 MPa and represents the satisfied cross-linking performance. This is more prominent in the Gly–AZC sample: the film keeps its original shape without any sign of swelling and the wet tensile stress reaches to 1.53 MPa, indicating that the co-cross-linking induces efficient linkages with hydroxyl groups of starch, and helps form a dense network that can limit the penetration of water molecules into the inner parts of starch films.

The surface hydrophobic properties of the cross-linked starch films are illustrated through the contact angle (CA) measurements. As presented in figure 7*c,d*, the initial water contact angle of control starch film (Control sample) is only 42.04° and decreases to 34.06° within the recording time (180 s), exhibiting a highly hydrophilic nature. The glyoxal cross-linked starch film almost doesn't improve the hydrophobicity compared with the control starch, and the initial water contact angle is only 50.91° and decreases to 41.27° at 11 s, then becomes stable until 180 s. Therefore, the cross-linking through glyoxal alone does not contribute to the improved surface hydrophobicity of starch. In contrast, the AZC starch film presents excellent hydrophobic properties with the initial CA of 99.45°. Besides, the water contact angle remains high and still over 90° (92.37°) at 180 s. The starch film co-cross-linked by glyoxal and AZC also exhibits improved surface hydrophobicity, compared with the Control sample.

### 3.6. Optical and mechanical properties analysis

**Figure 8.**
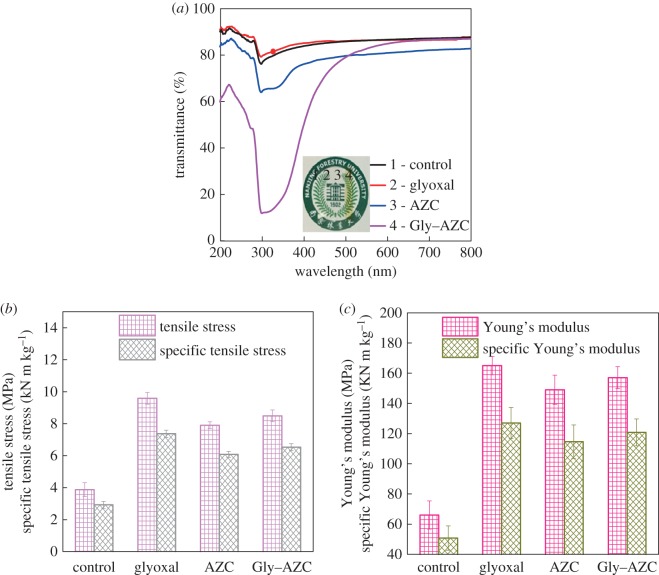
The UV–Vis transmittance (*a*), tensile stress and specific tensile stress (*b*) and Young's modulus and specific Young's modulus (*c*) of the starch films with the grammage of 140 g m^−2^.

The transmittance and optical properties of the control and cross-linked starch films are exhibited in figure 8. As shown, the Gly–AZC starch film presents excellent transmittance and the ‘logo’ image can be seen clearly. The cross-linking reaction gives an obvious visual colour difference in the Gly–AZC sample (yellow) compared with the other starch films (colourless and transparent). The yellow colour in the Gly–AZC sample is attributed to the presence of the Schiff-base structure. More importantly, the Gly–AZC sample still exhibits excellent transmittance at visible wavelength range.

Figure 8*a* depicts the light transmittance through the films as a function of wavelength. As seen, the transmittance at 600 nm is around 80% for these starch films except for the AZC sample. This might be attributed to the increased starch granule size due to the self-polymerization of AZC. For the Gly–AZC sample, the transmittance at 300 nm shows an obvious decrease, compared with other starch films. This is accorded with the result of UV–vis spectra in figure 1. The produced Schiff-base structure can adsorb the light at 300 nm and thus give a decrease to the transmittance correspondingly. It is noteworthy that the excellent UV shielding capacity between the 300 nm–330 nm ranges might be very beneficial for broadening the application fields of starch films in the packaging industry. The capacity for both excellent transmittance at a visible wavelength range and UV shielding capacity is extremely important for food packaging materials.

Tensile stress (*σ*) and the specific tensile stress (*σ*/*ρ*) of starch films are presented in figure 8*b*, where *ρ* is the density. The (*σ*/*ρ*) of Glyoxal film is 7.37 kN m kg^−1^ and increases by 152.24% compared with the Control sample. In AZC, it only increases by 108.22%. The difference might be attributed to the decreased amount of linking bonds in starch network cross-linked by AZC compared with the Glyoxal sample, as well as the relatively weaker hydrogen linkage in the AZC sample against the covalent bonding in the Glyoxal sample. For the Gly–AZC sample, the specific tensile stress (6.53 KN m kg^−1^) increases by 123.63%. Clearly, the strengthening effect of glyoxal is better than that of AZC at the same dosage. In fact, glyoxal has a lower molecular weight and a higher number of reactive groups that can form the covalent bonds with the hydroxyl groups of starch in comparison with AZC. Moreover, the weight ratio of glyoxal to AZC is also closely related to the strength performance of starch film cross-linked by the current novel co-cross-linking system, which has also been studied in subsequent works.

The relatively high modulus (E) of cross-linked starch films is shown in figure 8*c*. Glyoxal and AZC have been reported to improve the elastic modulus of cellulose-based materials. As expected, the addition of glyoxal and AZC gives higher tensile modulus than the control starch film. For better comparison, the specific tensile modulus (*E*/*ρ*) takes the density (*ρ*) variation into account. The *E*/*ρ* of Gly–AZC increases by 137.92% compared with the Control film. The increase ratio in the dual-cross-linking system is not as high as the Glyoxal sample but is higher than the AZC sample.

### 3.7. Morphology and topography of starch films

**Figure 9.**
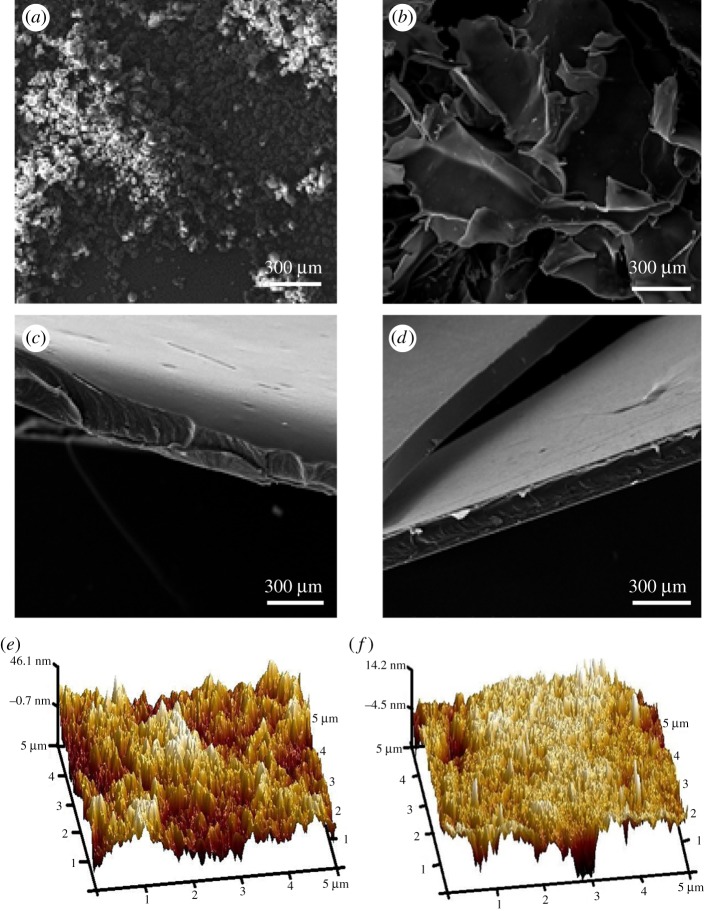
SEM surface images of starch granules: Control (*a*) and Gly-AZC (*b*). SEM fracture images of starch films: Control (*c*) and Gly-AZC (*d*). AFM topography images of starch films: Control (*e*) and Gly-AZC (*f*).

The SEM and AFM images of starch are shown in figure 9. As shown, the cross-linked starch after freeze-drying exhibits obvious different morphology compared with the Control sample. The starch granules after cross-linking present obvious connection, compared with the control starch, clearly visible in the SEM images (in figure 9*a–d*). As shown, the control starch granules are individual or separated with diameters of around 150 µm–300 µm (figure 9*b* and electronic supplementary material, figure S2a). However, the Glyoxal, AZC and Gly–AZC starch present a flaky shape, which is dramatically different to the un-cross-linked control starch granules (electronic supplementary material, figure S3a and S3b and figure 9*b*). Electronic supplementary material, figure S2 shows the cross-section images of starch films and there appear ‘hills and valleys’ in the fracture of Control and AZC film samples; while the cross-sections of the Glyoxal and Gly–AZC samples exhibit relatively smooth surfaces, indicating the increased rigidity after the co-cross-linking (electronic supplementary material, figure S2d and S2f). This change in the cross-section morphology after co-cross-linking can also be observed in figure 9*c,d*.

From the AFM images, it is clear that the surface of Gly–AZC starch film is more homogeneous and the height is only 14 nm, while the roughness of the control films is relatively higher. As seen in electronic supplementary material, figures S3c and S3d, the addition of glyoxal in Glyoxal film give a much more homogeneous surface than that of AZC film. This indicates that the addition of glyoxal efficiently limits the self-polymerization of AZC when co-cross-linked with starch and contributes to the formation of a uniform morphology in starch films.

## 4. Conclusion

In this assessing work, a starch film with enhanced hydrophobicity, strength and excellent transmittance at the visible light range and UV shielding ability was obtained via a novel co-cross-linking system. Based on the experimental findings, the following conclusions were achieved:

1. The addition of glyoxal and AZC significantly improved the hydrophobicity and strength of the co-cross-linked starch. The *E*/*ρ* of Gly–AZC increased by 137.92%, compared with the Control. The wet tensile stress reached to 1.53 MPa and the initial CA was 71.05°. Moreover, the excellent transmittance at visible wavelength range and UV shielding capacity were observed.
2. The UV, FT-IR spectra and ^13^C NMR confirmed the presence of Schiff-base reaction in the co-cross-linking system. XRD and TG analysis showed that the addition of glyoxal avoided the weakened crystallinity structure and the decreased MDT of co-cross-linked starch to some extent, via limiting the self-polymerization of AZC. SEM and AFM images indicated that a rigid and uniform starch film was formed after the co-cross-linking.

## Supplementary Material

Enhancing hydrophobicity, strength and UV-screen capacity of starch film via novel co-crosslinking

## Data accessibility

The datasets supporting this article have been uploaded as part of the electronic supplementary material.
