# Supplementary material for: Enhancing hydrophobicity, strength and UV shielding capacity of starch film via novel co-cross-linking in neutral conditions
Source: R Soc Open Sci. 2018 Nov 14;5(11):181206. doi: 10.1098/rsos.181206 (PMC6281899; doi:10.1098/rsos.181206)
Supplement: Enhancing hydrophobicity, strength and UV-screen capacity of starch film via novel co-crosslinking [file rsos181206supp1.docx]

**Enhancing hydrophobicity, strength and UV-screen capacity of starch film via novel co-crosslinking**

Shuzhen Ni ^a,c^ Liang Jiao ^a^ Hui Zhang ^b,c^ Yongchao Zhang ^d^ Guigan Fang ^a^ Huining Xiao ^c,^* Hongqi Dai ^a,^*

^a^ Jiangsu Co-Innovation Center of Efficient Processing and Utilization of Forest Resources, Nanjing Forestry University, Nanjing 210037, China

^b^ College of Materials Engineering, Fujian Agriculture and Forestry University, Fuzhou 350002, China

^c^ Department of Chemical Engineering, University of New Brunswick, Fredericton, New Brunswick E3B 5A3, Canada

^d^ Johan Gadolin Process Chemistry Centre, c/o Laboratory of Wood and Paper Chemistry, Åbo Akademi University, Turku FI-20500, Finland

**Fig. S1** (**a)** The UV-vis adsorption of glyoxal aqueous solution (2 %, wt), (**b)** the transmittance of 1 % AZC solution (1 %, wt).

The glyoxal and AZC aqueous solution are heated at 60 °C for 30 mins before the UV-vis analysis, respectively. As shown in Fig. S1 (a), the glyoxal aqueous solution is transparent without any change in color. By contrast, the AZC solution becomes turbid and its transmittance decreases significantly in the pH range of 4.5 to 9.5 (Fig. S1 b). It is ascribed to the decomposition of AZC resulting from the pH change upon heating (forming crystalline metal), which might trigger crosslinking.


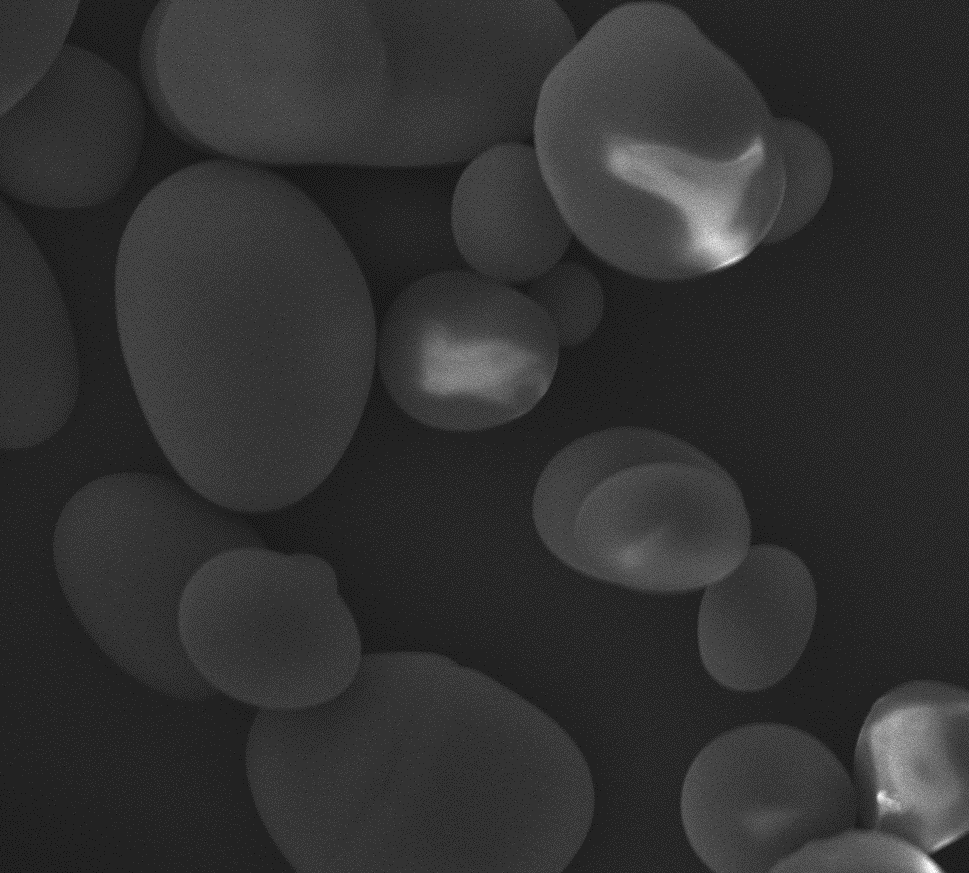

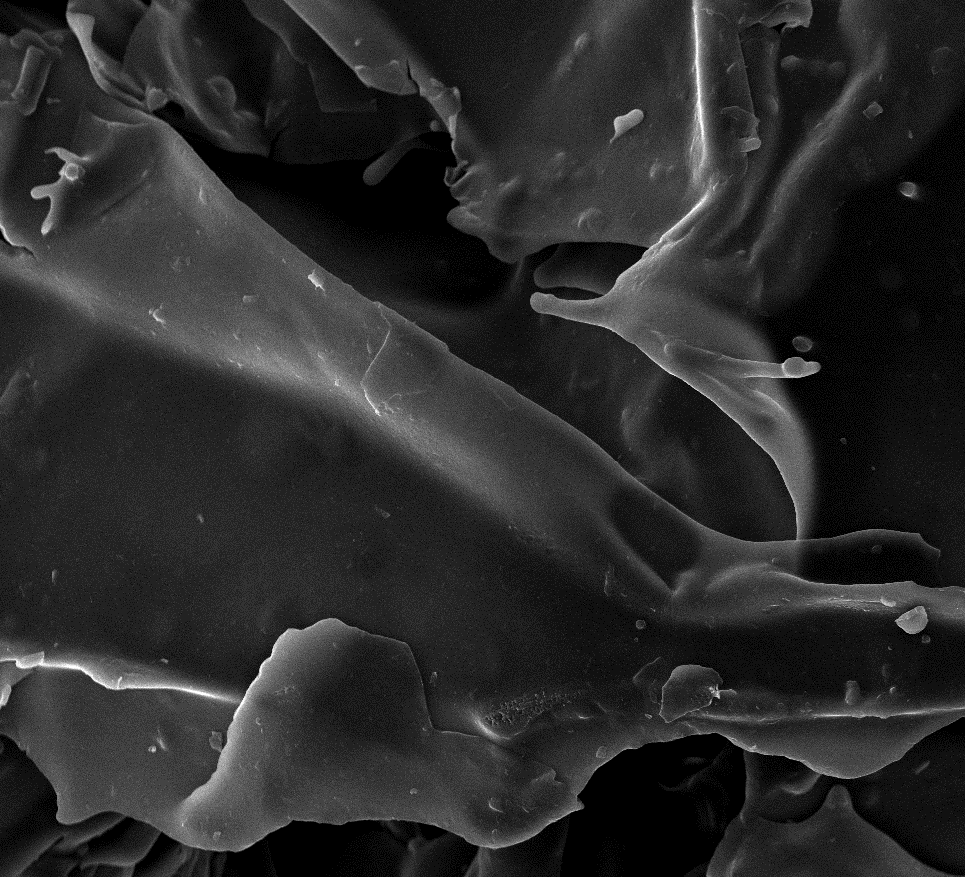


**40μm**

**40μm**

**(a)**

**(b)**


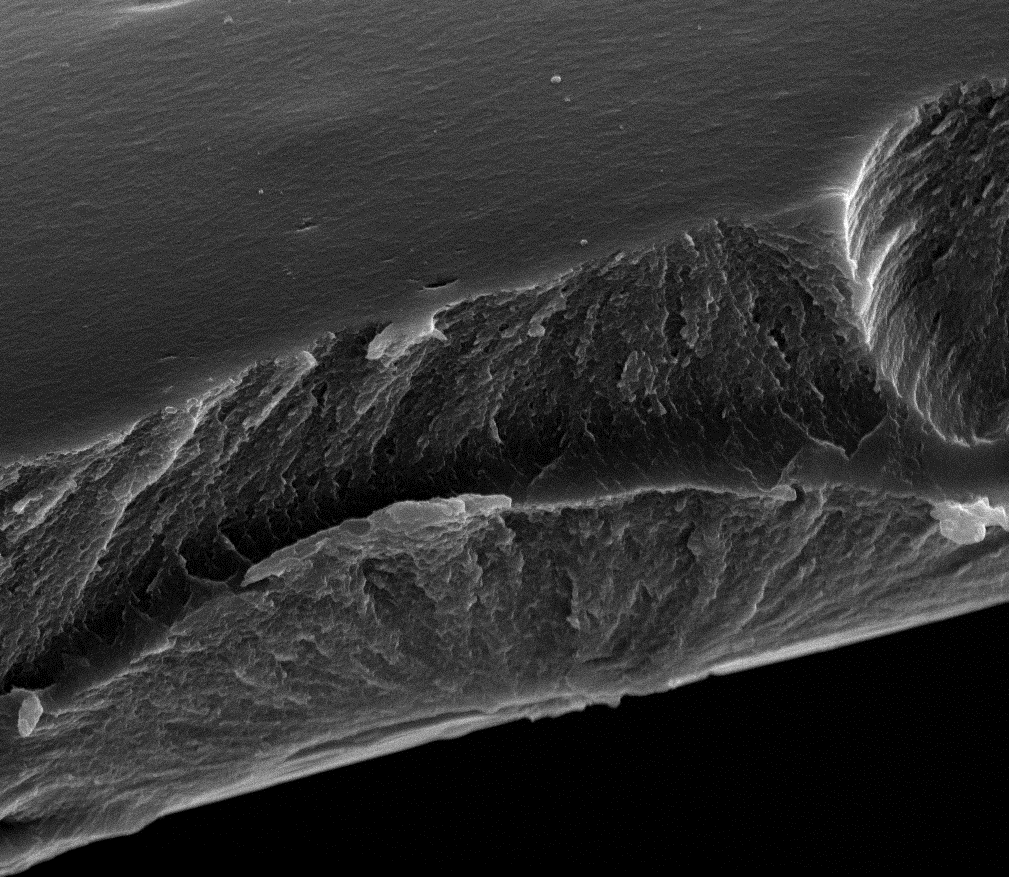

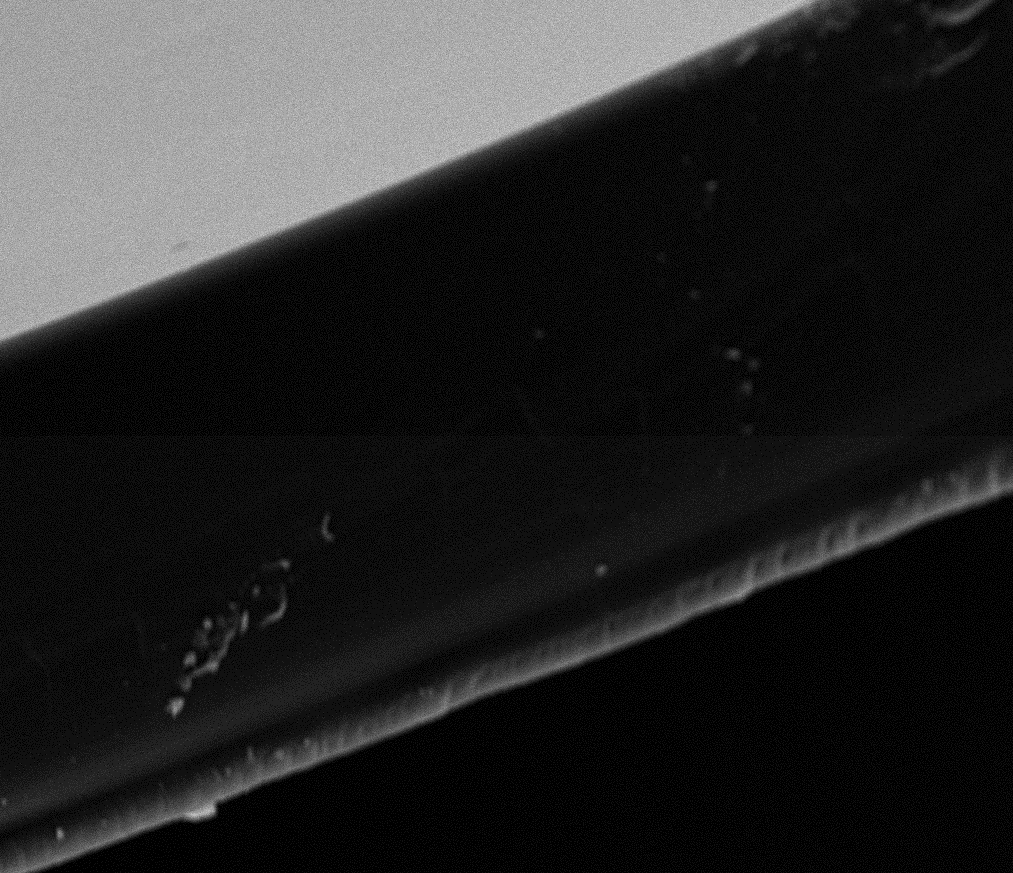


**(d)**

**(c)**

**(e)**

**(f)**

**40μm**

**40μm**


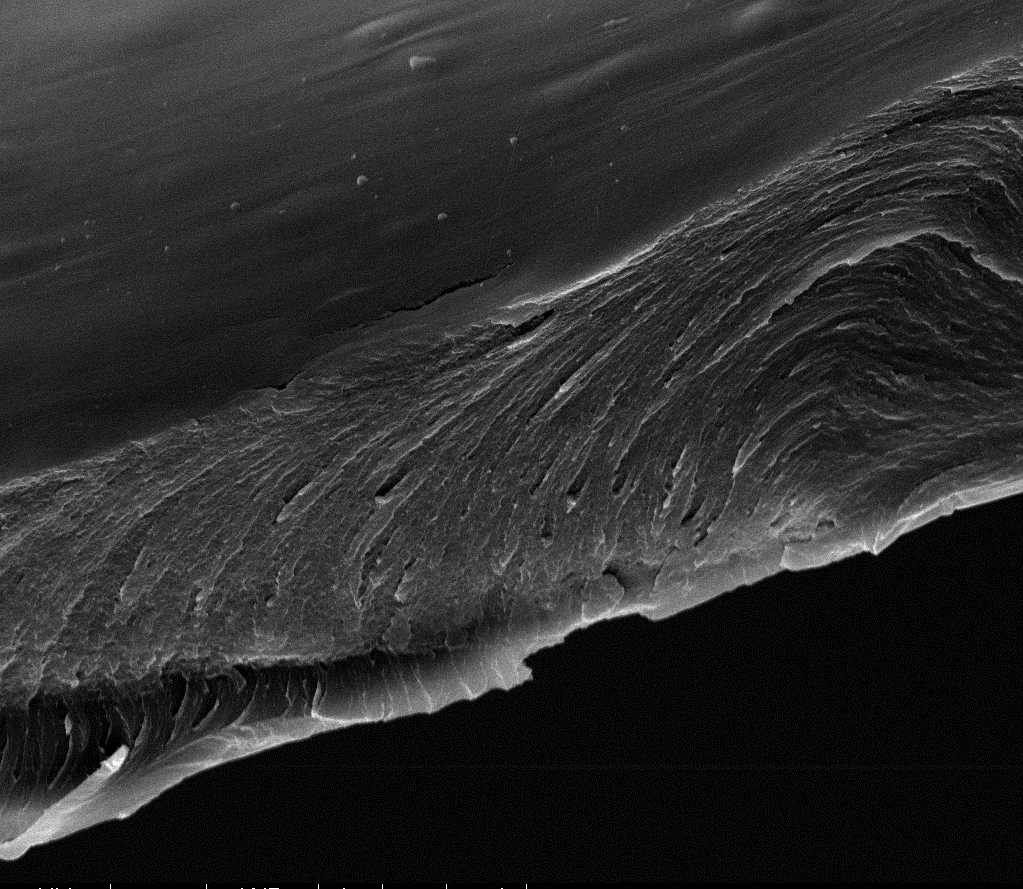

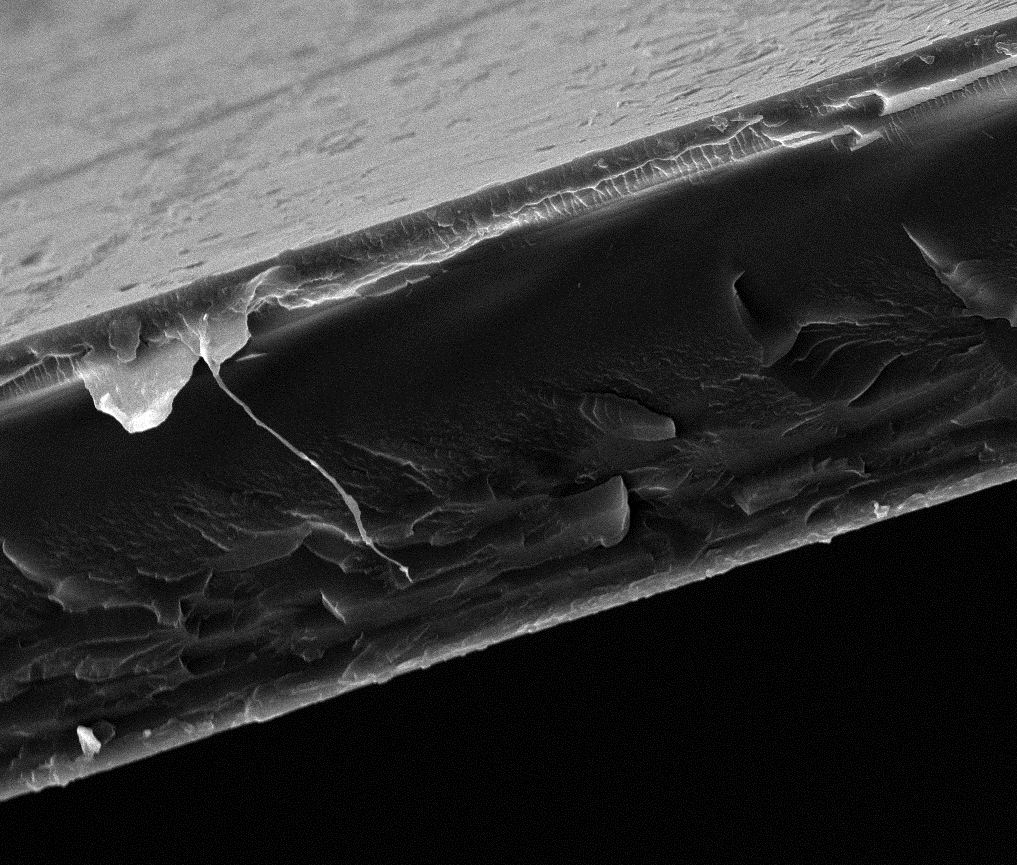


**40μm**

**40μm**

**Figure S2** SEM surface images of starch granules: Glyoxal (a) and AZC (b). SEM fracture images

**s**tarch films: Control (c), Glyoxal (d), AZC (e) and Gly-AZC (f).


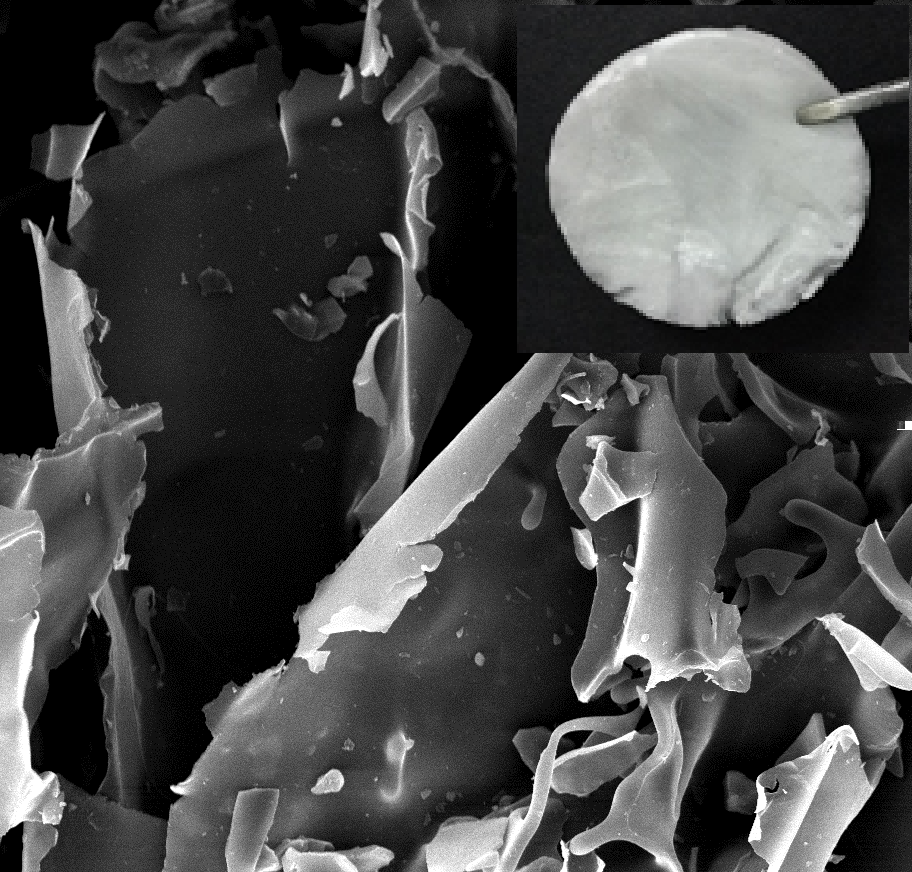

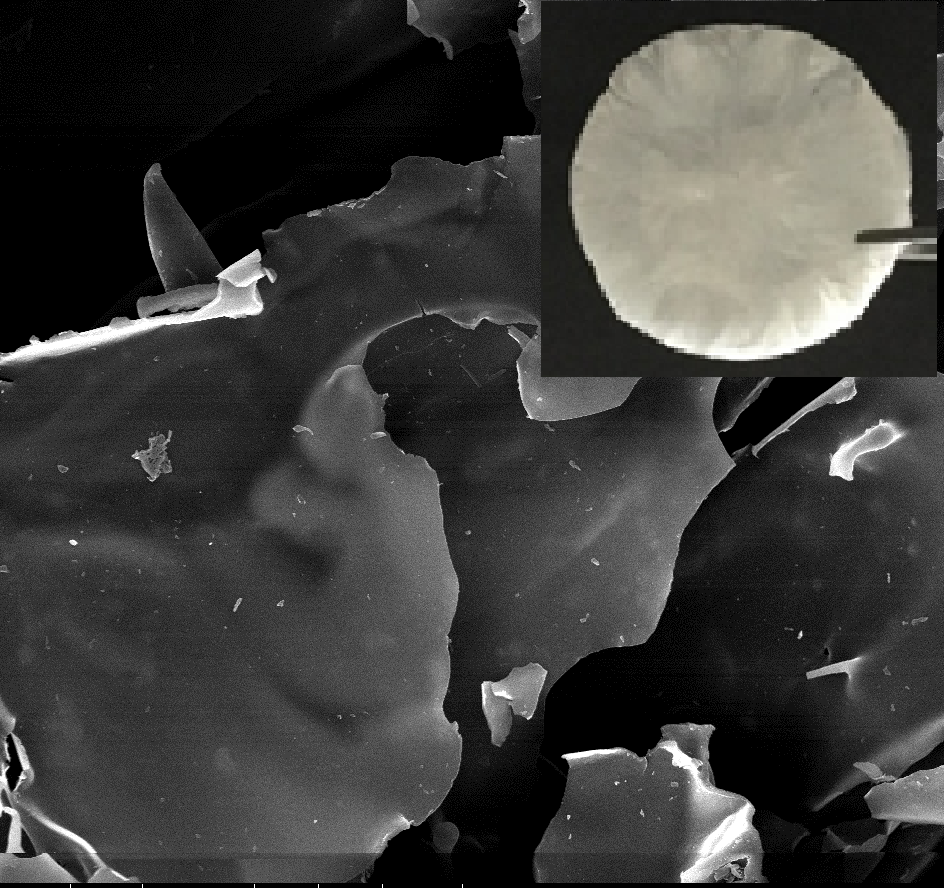


**(d)**

**(c)**

**300μm**

**300μm**

**(b)**

**(a)**


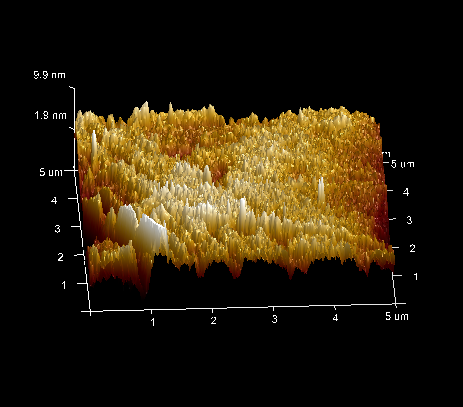

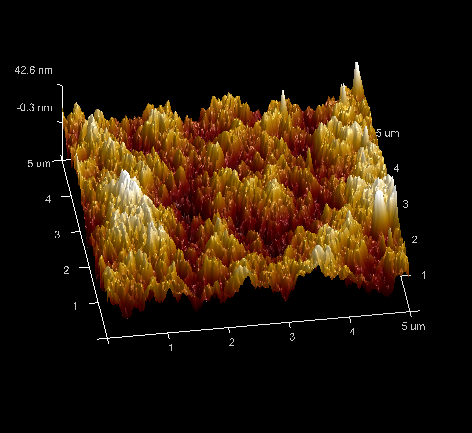


**300μm**

**300μm**

**Figure S3.** SEM surface images of starch granules: Glyoxal (a) and AZC (b). SEM fracture images of starch films: Glyoxal (c) and AZC (d). AFM topography images of starch films: Glyoxal (e) and AZC (f).
